# Complete genome sequences of *Arthrobacter globiformis* phages Uzumaki and Argan of cluster AU6

**DOI:** 10.1128/mra.00293-24

**Published:** 2024-07-11

**Authors:** Brandon Mathew, Andrew Sean Lee, Katie Chen, Michael Kaczmarski, Nigel Oommen, Asweel Mehaboob, Vrushali Patel, Yamini Patel, Hannah Saji, Muhammad Ayaan Shamsi, Bryan Gibb

**Affiliations:** 1 Department of Biological and Chemical Sciences, New York Institute of Technology, Old Westbury, New York, USA; 2 Department of Psychology and Neuroscience, University of Colorado, Boulder, Colorado, USA; Queens College Department of Biology, Queens, New York, USA

**Keywords:** bacteriophage, *Arthrobacter*, AU6, *Arthrobacter globiformis*, SEA-PHAGE

## Abstract

Bacteriophages Uzumaki and Argan infect *Arthrobacter globiformis* B-2880 isolated from soil samples in Long Island, New York. These bacteriophages have lambda-like morphology with prolate capsid and share 97% gene content similarity. These traits place them in cluster AU6 with other related *Arthrobacter* phages.

## ANNOUNCEMENT

**Fig 1:**
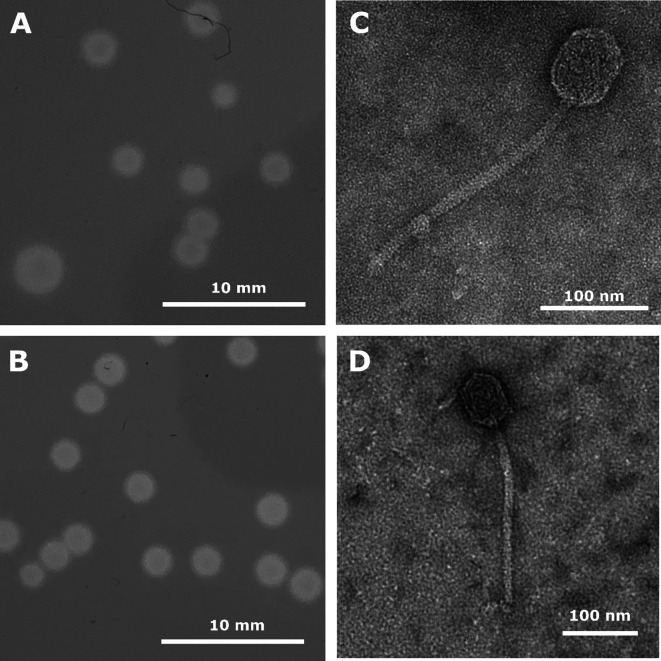
Plaque morphology (**A and B**) and transmission electron microelectron micrographs (**C and D**) of *Arthrobacter* phages Uzumaki (**A and C**) and Argan (**B and D**). Phage plaques were imaged following incubation at 30°C for 48 h. Phage lysate was negatively stained with 1% uranyl acetate and imaged with a JEOL JEM-1400 transmission electron microscope at 120 KeV.

*Arthrobacter* phages Argan and Uzumaki were isolated from moist soil samples collected in Glen Oaks and Elmont, New York, respectively. Phage isolation, plaque purification, and genome extraction were performed according to the standard procedures described in the SEA-PHAGES Discovery Guide (https://seaphagesphagediscoveryguide.helpdocsonline.com/home), as part of the Science Education Alliance-Phage Hunters Advancing Genomics and Evolutionary Science (SEA-PHAGES) program (1). Both Argan and Uzumaki were isolated by enrichment on *Arthrobacter globiformis* B-2880 utilizing a peptone–yeast–calcium (PYCa) medium at 30°C for 24–48 h, undergoing three rounds of plaque purification and form clear round plaques 2–6 mm in diameter with defined edges (Fig. 1). High-titer phage lysate was produced by plate lysis using double-agar overlays in phage buffer and 0.22 μm filtered. Negative-stained transmission electron microscopy showed that both phages have prolate icosahedral heads (58–64 nm) and (236–238 nm) long non-contractile tails, consistent with other Caudoviricetes bacteriophage-like phage-lambda (Fig. 1) (2, 3).

Genome extraction was performed from high-titer lysates using the Wizard DNA cleanup kit (Promega), and sequencing was performed at the University of Pittsburgh. Libraries were constructed using the NEB Ultra II FS DNA library prep kit and sequenced using an Illumina MiSeq v3 platform with single-end sequencing, resulting in 150 bp reads. Raw reads were assembled using Newbler v2.9 (4) with default settings, generating single contigs with an average read depth of approximately 1,250 for Uzumaki and 441 for Argan. The phage contigs were verified using Consed v29 to evaluate completeness and determine genomic termini (5). The genome parameters (length, GC content, and termini) and accession numbers (GenBank and SRA) are shown in Table 1. The genomes of both phages have defined ends with nine-base complementary 3′ single-stranded extensions.

**TABLE 1:** Phage GenBank and SRA accession numbers and genome assembly results

Phage	Genbank accession no.	SRA accession no.	Location (GPS coordinates)	Avg coverage	No. of reads (thousands)	Genome size (bp)	Gc %	Genome end (3' overhang)	No. of genes
Argan	OR613480.1	SRX20165760	40.74181 N, 73.71456 W	441	650.85	55,220	50.3	CGCCGGCCT	92
Uzumaki	ON970608.1	SRX14485100	40.70616 N, 73.70895 W	1250	486.77	55,355	50.4	CGCCGGCCT	92

Both phages were assigned to cluster AU6 containing other related *Arthrobacter* phages based on the gene content similarity (GCS) greater than 35% using the tool at PhagesDB (6, 7). Coding regions were predicted using GeneMark v3.25 (8) and Glimmer v3.02 (9). The coding regions were manually checked using Starterator v1.2 (http://phages.wustl.edu/starterator/), PECAAN v20211202 (https://blog.kbrinsgd.org/), and DNA Master v5.23.6 (10). Functions for the genes were found using NCBI BLASTp v2.9 (11), HHPred v2.0.13 (12), and Phamerator (13). No tRNA genes were identified by Aragorn v1.2.41 (14) and tRNAscanSe v2.0 (15). The membrane proteins were predicted using DeepTmHmm v2.0 (16) and TOPCONS 2.0 (17). All software was used with default settings.

Uzumaki and Argan are both predicted to encode 92 protein-coding genes, but only 25 and 23 are assigned putative functions. Argan and Uzumaki have 97% nucleotide identity and 85.87% GCS (6, 7). All genes in both genomes are transcribed on the same strand. The genome organization for both phages is similar, with the genes coding for phage structure and assembly located on the left half of the genome, whereas the genes involved in DNA replication and metabolism are found on the right half. No integrase or immuno-repressor genes were identified, suggesting that the phage exhibits an entirely lytic cycle, which is consistent with the clear plaque morphologies observed in each phage.

## Data Availability

Genbank accession and Sequence read archive (SRA) numbers for phages Uzumaki and Argan are provided in Table 1.
